# Editorial: Infectious Disease Epidemiology and Transmission Dynamics

**DOI:** 10.3390/v15010246

**Published:** 2023-01-15

**Authors:** Zhanwei Du, Wei Luo, Rachel Sippy, Lin Wang

**Affiliations:** 1 WHO Collaborating Center for Infectious Disease Epidemiology and Control, School of Public Health, LKS Faculty of Medicine, The University of Hong Kong, Hong Kong SAR, China; 2 Laboratory of Data Discovery for Health Limited, Hong Kong Science Park, Hong Kong SAR, China; 3 Department of Geography, National University of Singapore, Singapore 117570, Singapore; 4 Saw Swee Hock School of Public Health, National University of Singapore, Singapore 117570, Singapore; 5 Department of Genetics, University of Cambridge, Cambridge CB2 3EH, UK

Infectious diseases, such as COVID-19 [1], influenza [2], dengue [3], and monkeypox [4], have caused significant burdens to population health and socioeconomic development in the world. Scientists have unraveled many aspects as to the transmission dynamics and population health strategies to mitigate the spread of epidemics [5,6,7,8,9,10,11,12,13,14,15]. This Special Issue contains eleven original articles and two commentaries for scientific endeavors that bring together expertise and efforts toward this common goal.

The research includes studies on the reproduction number of SARS-CoV-2 variants, vaccine efficacy, antiviral efficacy, and the epidemiological impact of nonpharmaceutical interventions. Our research mainly focuses on COVID-19, an urgent problem to solve in 2022 with the emergence of multiple SARS-CoV-2 variants that can escape human immunity elicited by previous infection or vaccination [16,17]. Du et al. and Jin et al. reviewed and estimated the reproduction number of SARS-CoV-2 variants (e.g., Omicron, Delta) to evaluate their transmission advantages [16,18] and found that asymptomatic spreaders could be identified from the transmission network [19]. Mass vaccination, treatment, and mass testing can help reduce the overall population-level attack rate and prove critical to early pandemic mitigation. Wang et al. assessed the epidemiological impact of vaccination on COVID-19 in Hong Kong for the ancestral, Delta, and Omicron strains [20]. The mRNA vaccines are useful for reducing the risk of death for moderate cases but are not significant for severe cases [21]. Koo et al. estimated that fortnightly and weekly mass routine rapid antigen testing would reduce overall infections by 12.8% and 25.2%, respectively [22]. The 2021-22 cross-sectional study using a self-administered questionnaire in Algeria suggested that the impact of preventive measures and vaccination against SARS-CoV-2 is statistically significant in reducing the risk of infection, treatment, and hospitalization [23].

Alongside the studies on COVID-19, several papers also analyzed other pathogens. Zhang et al. tested the structural identifiability of four humidity-driven epidemiology models of influenza transmission [24]. Espitia et al. analyzed the mathematical model of HIV/AIDS presented by Espitia to reveal that reducing homosexual partners can reduce contagion and consequently reach a DFE [25]. Ito et al. and Loi et al. learned about the transmission dynamics of African swine fever in wild boars in South Korea and Italy [26,27]. Soh et al. also proposed a mechanistic model to study the epidemiological impact of fertile Wolbachia-infected female mosquitoes from being released into the environment [28].
